# HUMERAL SHAFT FRACTURE WITH AN INTACT WEDGE FRAGMENT: MIPO VS CONVENTIONAL PLATING

**DOI:** 10.1590/1413-785220233103e268121

**Published:** 2023-09-08

**Authors:** Jorge Henrique Higashi, Felipe Cruz Caetano dos Reis, Caio Filipe Antunes Guimarães, Ricardo Debussulo de Lima, Fernando Brandao Andrade-Silva, Jorge dos Santos Silva, Kodi Edson Kojima

**Affiliations:** 1 Universidade de Sao Paulo, Faculdade de Medicina, Hospital das Clinicas HC-FMUSP, Instituto de Ortopedia e Traumatologia IOT, Grupo de Trauma, Sao Paulo, SP, Brazil.; 2 Universidade José do Rosário Vellano, Alfenas, MG, Brazil.; 3 Santa Casa de Misericórdia de São Sebastião do Paraíso, São Sebastiao do Paraiso, MG, Brazil.

**Keywords:** Humerus, Diaphysis, Bone Consolidation, Complications, Úmero, Diáfise, Consolidação Óssea, Complicações

## Abstract

**Objectives:**

Evaluate bone healing time, consolidation, and the complication rate between the minimally invasive plate osteosynthesis and open reduction with plate osteosynthesis in humeral diaphyseal fractures with an intact wedge (AO 12B2).

**Methods:**

A retrospective study was carried out between 2016 and 2020. The medical records and radiographs of 18 patients were analyzed, and data were collected regarding the time of consolidation, age, sex, plate size, number of screws, complications such as iatrogenic injury damage to the radial nerve, material failure, and postoperative infection.

**Results:**

No statistically significant differences were observed in the variables of age, sex, plate size, and number of screws used or in the RUSHU index (Radiographic Union Score for Humeral fractures). There were no postoperative infections, material failure, or need for reoperation, nor cases of secondary radial nerve injury. After one year, all patients had a consolidation index analyzed by RUSHU >11.

**Conclusion:**

both techniques showed similar results, with a high consolidation rate and low rates of complications or iatrogenic damage to the radial nerve. ***Evidence level III; Retrospective comparative study*** .

## RESUMO

**Objetivos:**

Comparar o tempo de consolidação e o índice de complicações entre os métodos de osteossíntese com placa minimamente invasiva e estabilidade absoluta através da placa nas fraturas diafisárias do úmero com cunha intacta (AO 12B2).

**Métodos:**

Foi realizado um estudo retrospectivo entre os anos de 2016 e 2020. Foram analisados os prontuários e radiografias de 18 pacientes e coletados dados referentes a: tempo de consolidação, idade, sexo, tamanho da placa, número de parafusos, presença de complicações como lesão iatrogênica do nervo radial, falha do material e infecção pós operatória.

**Resultados:**

Não foram observadas diferenças estatisticamente significativas nas variáveis de idade, sexo, tamanho da placa e número de parafusos utilizados, ou no índice de RUSHU (Radiographic Union Score for Humeral fractures). Não houve casos de infecção pós-operatória, falha do material ou necessidade de reoperação, nem casos de lesão secundária do nervo radial. Após 1 ano todos os pacientes tiveram índice de consolidação analisado pelo RUSHU >11.

**Conclusão:**

Ambas as técnicas se mostraram com resultados similares, com alta taxa de consolidação e baixas taxas de complicações ou lesão iatrogênica do nervo radial. ***Nível de evidência III; Estudo retrospectivo comparativo*** .

## INTRODUCTION

Humeral fractures account for 5% to 8% of all fractures, and the shaft segment comprises approximately 20% of the humeral fractures and 3% of all long bone fractures.^1 , 2^

Besides the historically used conservative treatment, many surgeons tend to prefer the operative treatment based on the reported nonunion rate, residual deformity, and joint stiffness.^3 – 5^ Currently the open reduction with plate osteosynthesis (ORPO) remains the gold standard for the operative treatment,^6 , 7^ which has the advantage of the anatomical reduction, early range of motion, high rate of bone healing and possibility to explore and visualize the radial nerve.^8 , 9^ In the other hand to minimize the extensive dissection of the ORPO the minimally invasive plate osteosynthesis (MIPO) has emerged as a procedure which preserves the soft tissue envelope and periosteal circulation.^10 , 11^ Intramedullary nailing (IMN) is also another less invasive technique, but recent studies have reported high rates of re-operation and insertion site morbidity.^12 , 13^

The humeral shaft fractures are classified according to the Arbeitsgemeinschaft für Osteosynthesefragen (AO) / Orthopaedic Trauma Association (OTA) combined classification^14^ in simple type fractures (A), fractures with wedge fragment (B) and complex (C). In simple type A fractures Kim et al. have shown that MIPO is equivalent to ORPO as a safe and effective method of fixation.^15^ Jiang et al. and Livane and Belangero have published better results with the MIPO for comminuted fractures.^10 , 16^

In type B shaft fractures with intact wedge is not clear whether is better to do the ORPO technique to achieve absolute stability or MIPO technique to achieve relative stability.

The incidence of type B shaft fractures is around 29% of the humeral shaft fractures,^1^ causing possible limitation in the number of patients to be included, leading the authors to a more modest goal: to evaluate the difference in healing and complication rate between the ORPO and MIPO for the treatment of the AO/OTA 12B2 type fractures.

## PATIENTS AND METHODS

This retrospective study was performed at an urban university-based level 1 trauma center, between 2016 and 2020. Data were collected through a retrospective chart review and review of existing radiographs. Ethical approval was provided by the Scientific and Ethical Committee of the university under the protocol 52567121.5.0000.0068. Written informed consent was obtained from all included patients.

The inclusion criteria were as follows: humeral shaft fractures (5 cm bellow the surgical neck and 5 cm above the olecranon fossa) with an intact wedge fragment (AO/OTA 12B2), with less than two weeks, an age older than 16 years with completion of growth, signed informed consent and at least 12-month follow-up with all necessary radiographs for the healing assessment.

The exclusion criteria included fractures with more than two weeks, AO/OTA types A and C, open fractures Gustilo type IIIB and C, any treatment other than plate fixation with ORPO or MIPO, pathologic fracture, refracture, proximal and distal humeral fractures, and incomplete follow-up.

Demographic data on the following were collected: age, sex, mechanism of trauma, associated injuries, primary radial nerve injury, AO/OTA classification, and Gustilo classification ^17^ for open fractures.

In the ORPO group were included all the fractures where the intact wedge fragment was anatomically reduced, interfragmentary compression achieved and a rigid fixation applied following the AO principle of absolute stability.^19^ In the MIPO group were included shaft fractures where indirect reduction was applied correcting the alignment, length and rotation and the fixation was done with long plates and a flexible construct.^18^

The data relative to the surgical procedure collected were the length of the plate, number of screws in each side of the fracture, fixation working length and presence or not of a lag screw.

The variables collected in the follow up were secondary radial nerve injury, infection as defined by Metsemakers et al.^19^ , and bone healing using the RUSHU (Radiographic Union Score for Humeral fractures) ^20^ . In this score each cortex (anterior, posterior, lateral and medial) receives points from one to three, based on the healing stage, being one point for absence of callus formation, two points for presence of a non-bridging callus and three points for a bridging callus. A score less than seven points was considered nonunion (≥ 8 was considered healed).

Descriptive statistics included means and standard deviations for continuous variables and counts (percentages) for categorical variables. Statistical analysis of infection and nonunion was performed using the chi-square test or Fisher's exact test. Comparative analysis was performed according to the outcome and compared using Student's t-test. Odds ratios were estimated with the respective 95% confidence intercal and adjusted with the model of multiple logistic regression with the variables that presented with a descriptive level of bivariable analysis less than 0.10 (p<0.10). Statistical analysis was performed using IBM SPSS software for Windows version 22.0, with a significant level of 5%.

## RESULTS

A total of 93 patients with humeral shaft fracture were treated between 2016 and 2020, and we could get data from 66 patients, because 26 lost follow-up and did not complete the one-year follow-up and one patient died due to cause nonrelated to the fracture. After applying the inclusion and exclusion criteria, 18 patients (27.2%) were included. The group was composed by 7 (38.9%) men and 11 (61.1%) women, with a mean age of 45.1 years. Of the 18 patients, 10 (55.5%) were treated with open reduction and plate osteosynthesis (ORPO) and the remaining 8 (44.5%) were treated with minimally invasive plate osteosynthesis (MIPO). There was no significant difference in age(p = 0.911) and gender (p = 0.802) between the ORPO and MIPO groups ( Table 1 ).

**Table 1:** Demographic data.

	ORPO (n= 10)	MIPO (n=8)	p
Mean age	45.2 ± 17.5	45.0 ± 13.2	0.911
Sex			0.802
Female	6 (60%)	5 62.5%)	
Male	4 (40%)	3 37.5%)	
Open fracture	1 (10%)	2 (25%)	0.512
Primary injury radial nerve	2 (20%)	3 (37.5)	0.476

In the ORPO group, one patient (10%) had open fracture, compared with two patients in the MIPO group (25%). Primary injury of the radial nerve occurred in two patients (20%) in the ORPO group and in three (37.5%) in the MIPO group. Both parameters showed no statistical difference between the two groups, respectively p = 0.512 and p = 0.476 ( Table 1 ).

The plate length was defined by the number of screw holes of the plate and in the ORPO group the average length was 9.4 ± 1.3 (7 – 12) holes and in the MIPO group 11.8 ± 2.0 (10 – 16) holes, showing a significant difference between the groups (p=0.009). The average number of screws in each side of the plate did not have statistical difference between the groups, being 3.5 ± 0.5 screws in the ORPO group and 3.0 ± 0.8 screws in the MIPO group ( Table 2 ).

**Table 2:** Plate and screws.

	ORPO (n= 10)	MIPO (n=8)	p
Mean plate length	9.4 ± 1.3 (7 – 12)	11.8 ± 2.0 (10 – 16)	0.009*
Number of screws in each side of the plate	3.5 ± 0.5 (3 – 4)	3.0 ± 0.8 (3 – 4)	0.118
Infection (superficial / deep)	0 (0%)	0 (0%)	
Plate failure	0 (0%)	0 (0%)	
Reoperation	0 (0%)	0 (0%)	
Secondary radial nerve injury	0 (0%)	0 (0%)	
Mean RUSHU (6m)	10.6 ± 2.3	9.1 ± 2.9	0.247
Mean RUSHU (12m)	11.4 ± 8.4	11.5 ± 0.7	0.798

RUSHU - Radiographic Union Score for Humeral fractures.

There was no infection, nor plate failure or reoperation in any of the groups. There was also no secondary radial nerve injury in neither group.

The mean RUSHU in the six-month follow-up was 10.6 ± 2.3 in the ORPO group, with one case with RUSHU > 7. In the MIPO group it was 9.1 ± 2.9, with three patients with score > 7. There was no statistically significant difference between the groups ( Table 2 ).

The 12-month follow-up RUSHU was 11.4 ± 8.4 in the ORPO group and 11.5 ± 0.7 in the MIPO group (p=0.798). All patients had score > 11 in both groups ( Table 2 ).

## DISCUSSION

Fractures of humeral shaft is defined as the segment distal to the surgical neck and proximal to the epicondyles and make up 5 to 8% of all fractures [1,2]. The most common fracture type is type A (simple, including spiral, oblique, or transverse fractures), followed by type B (including intact wedge or fragmented wedge) and type C (complex, including segmental or complex).^21^

Historically nonoperative treatment with functional brace has been used, however, due to the high rate of non-union, residual deformity and joint stiffness many orthopedic surgeons tend to prefer the operative treatment^3 , 22^ , particularly in severely displaced, comminuted, or segmented; demands for improved functional results and earlier rehabilitation.^23^

Operative treatment options include plate fixation or intramedullary nailing. Fixation with intramedullary nail has biomechanical advantages and good rates of bone healing, but recent studies have reported higher rates of reoperation and insertion site morbidity when compared to plate fixation, thus, plate fixation is considered gold standard for operative treatment.^24 , 25^

Fixation with plate can be done with absolute stability with anatomical reduction, interfragmentary compression and rigid fixation, also known as open reduction and plate fixation (ORPO), which has the advantage of multiple surgical approach, possibility to explore the radial nerve and a perfect reduction of the fracture and but the disadvantage of potential higher risk of infection, non-union and secondary injury to the radial nerve caused by the more extensive soft tissue dissection and periosteal blood supply.^15^

Plate fixation can also be done with a minimally invasive technique (MIPO) with functional reduction and flexible fixation.^18 , 19^ This bridge plate technique has the potential to minimize the complications due to smaller incisions and the percutaneous method to insert and fix the plate.^15 , 26^

Following the mechanical thinking simple type fracture due to high strain would do better with ORPO and on the other hand multifragmented fractures with low strain would do better with MIPO.

Nevertheless, Kim et al. (2015) ^15^ have done a prospective randomized study to compare ORPO and MIPO applied in simple type fracture of the humerus and found equivalent overall union rate and excellent functional outcomes in both groups.

Wang et al (2015) ^23^ focused their study on the evaluation of the comparison of the malrotation and functional results of the MIPO technique and ORPO. Both groups exhibited satisfactory union results and final shoulder function scoring. A significant incidence of malrotation (> 20°) was observed in the MIPO group (40.9% vs. 0%; p < 0.01). The malrotation significantly impacted the range of motion of the shoulder.

Esmailiejah et al. (2015)^28^ in a prospective randomized study with 68 patients, have found a shorter median time to union in the MIPO group (4 months vs. 5 months). Varus deformity > 5° was more common in the MIPO group (18.7% vs. 6.0%). There haven't found significant difference in the functional result and complications (infection, non-union and iatrogenic radial nerve injury).

Hu et al. (2016) ^27^ in a meta-analysis did not detect any significant difference in terms of operative time, fracture union rate, and fracture union time. The total complication rate was 20.1% in the ORPO group, compared with 5,1% in the MIPO group with a statistically significant difference (p<0.01). The main factor impacting this difference was the rate of iatrogenic radial nerve palsy that was lower in the MIPO (2.2% vs 10.4%).

All these studies analyzed all types of shaft fracture, including simple (type A), wedge (type B) and complex (type C) fractures. To our knowledge this is the first study to compare ORPO and MIPO in humeral fractures with intact wedge fragment (type B2).

The presence of an intact wedge allows the surgeon to opt for the absolute stability because it is possible to anatomically reduce the wedge and produce interfragmentary compression with lag screws. The concern is the dissection needed to manipulate the wedge fragment if this can affect the biology enough to impair the healing rate or to produce higher complication rate like infection and reoperation.

With the MIPO technique usually the reduction is indirect and closed, preserving the fracture hematoma. Care should be taken to have the wedge fragment close enough to the main fragments to have its healing.

The plate length was shorter in the ORPO group than in the MIPO group (mean 9.4 holes vs. 11.8 holes). In the ORPO the plate to be shorter was expected because with the open reduction the tendency is to use the shortest place possible to avoid long incisions, the plate should have enough length to bridge the area of the wedge and have three screws in each side of the fracture. To avoid invading the fracture hematoma in the MIPO plate the surgical incisions are placed more proximal and distal, thus, the need for a longer plate. Shorter plates have a shorter leaver arm and because of this the need for more screws, the longer the plate less screws are needed, this explain why in the ORPO the mean number of screws were higher than in the MIPO (3.5 vs. 3.0).

The mean RUSHU with 6 months didn't show a statistically significant difference between the two groups and were higher than 8, the threshold to consider the fracture healed (10.6 vs. 9.1; p=0.247). Although one can consider all healed, analyzing the absolute number of cases with RUSHU < 8 in each group, the results show 1 case out of 10 in the ORPO group, and 3 cases out of 8 in the MIPO group. This difference might be explained by the fact that a well done ORPO heals primarily without callus formation, so it is easier to interpret the x-ray as having higher RUSHU score.

After one-year follow-up all fractures were healed in both groups, all having RUSHU score 11 and 12 (mean 11.4 vs. 11.5; p=0.798), without any reoperation or intervention. Implant failure was also zero in both groups.

The iatrogenic secondary radial nerve injury was also absent in both groups. This shows that both methods are safe if done properly. In the ORPO a careful dissection and exposition of radial nerve must be done in all procedures and car should be taken to protect it all the time. With the MIPO the radial nerve is not dissected, but the anterior placement of the plate is safe for the nerve.

There was no superficial or deep infection in both groups.

The main limitation of this study is the number of included patients (18), ten in the ORPO and 8 in the MIPO group. This can be explained by the fact that the humeral shaft fracture is not as common as lower extremity fractures and the study addressed only a subgroup of those fractures, only humeral shaft fractures with an intact wedge (OA/OTA 12B2), which represents less than 30% of the humeral fractures. ^1^ This low number of patients influenced the statistical analysis. A larger number of patients could provide more information to validate the results. Radiographic analysis has always a subjectivity when giving score in the RUSHU method. Another limitation is to only have radiographic evaluation without a functional result.

In conclusion, the study shows that ORPO and MIPO have similar results in the surgical treatment of the humeral shaft fractures with an intact wedge, with high healing rates and low complications, including infection and iatrogenic radial nerve injury.

## CONCLUSION

In the surgical fixation of humeral shaft fracture with intact wedge (AO/OTA 12B2), open reduction and plate fixation (ORPO) produces similar result as minimally invasive plate osteosynthesis (MIPO), with high healing rates assessed by the RUSHU score and low infection and iatrogenic secondary radial nerve injury.
